# Robustness and reproducibility of an artificial intelligence‐assisted online segmentation and adaptive planning process for online adaptive radiation therapy

**DOI:** 10.1002/acm2.13702

**Published:** 2022-07-07

**Authors:** John W. Chapman, Dao Lam, Bin Cai, Geoffrey D. Hugo

**Affiliations:** ^1^ Radiation Oncology Washington University School of Medicine St. Louis Missouri USA; ^2^ Radiation Oncology University of Texas Southwestern Medical Center Dallas Texas USA

**Keywords:** adaptive radiotherapy, Ethos, quality assurance

## Abstract

Clinical implementation of online adaptive radiation therapy requires initial and ongoing performance assessment of the underlying auto‐segmentation and adaptive planning algorithms, although a straightforward and efficient process for this in phantom is lacking. The purpose of this work was to investigate robustness and repeatability of the artificial intelligence‐assisted online segmentation and adaptive planning process on the Varian Ethos adaptive platform, and to develop an end‐to‐end test strategy for online adaptive radiation therapy. Five synthetic deformations were generated and applied to a computed tomography image of an anthropomorphic pelvis phantom, and reference treatment plans were generated from each of the resulting deformed images. The undeformed phantom was repeatedly imaged, and the online adaptive process was performed including auto‐segmentation, review and manual correction of contours, and adaptive plan creation. One adaptive fractions in five different deformation scenarios were performed. The manually corrected contours had a high degree of consistency (> 93% Dice similarity coefficient and < 1.0 mm mean surface distance) across repeated fractions, with no significant variation across the synthetic deformation instance except for bowel (*p* = 0.026, one‐way ANOVA). Adaptive treatment plans also resulted in highly consistent dose–volume values for targets and organs at risk. A straightforward and efficient process was developed and used to quantify a set of organ specific contouring and dosimetric action levels to help establish uncertainty bounds for an end‐to‐end test on the Varian Ethos system.

## 1 INTRODUCTION

Adaptive radiation therapy (ART) is the process of adjusting the treatment plan in response to daily anatomy changes. When ART occurs immediately prior to treatment delivery without moving the patient, the process is called “online ART”. Studies have shown promising results for improving outcomes with online ART for several disease sites.^1^ Currently, online ART can be carried out based on magnetic resonance imaging, computed tomography (CT), and cone beam computed tomography (CBCT).^2^ An example is the recently available Varian Ethos system (Varian Medical Systems, Palo Alto, CA), which offers the flexibility to adapt to changing patient anatomy in the online setting based on CBCT images. This platform utilizes a highly integrated workflow with artificial intelligence (AI) tools to assist with the online segmentation and adaptive planning process of ART with the goal of improving consistency and efficiency in these processes. The AI models are based on convolutional neural networks and are designed, trained, and validated by the vendor. Training is done in supervised learning setting with ground‐truth contours derived from several hundred patients collected from multiple clinics. As is common for deep learning‐based autosegmentation algorithms, a cost function is constructed and iteratively optimized to reduce the errors between predicted value and ground truth value.

Prior to clinical deployment, the robustness and reproducibility of the AI‐assisted online ART process needs to be evaluated.^3^ There have been several studies evaluating performance of the Ethos system on clinical data. Sibolt et al. evaluated adaptive planning and AI‐assisted contouring for 39 pelvic cases using a pre‐clinical emulator of the Ethos software.^4^ Byrne et al. evaluated the need for editing of AI‐generated contours for intact prostate and prostate bed and the quality of adaptive plans generated online.^5^ Moazzezi et al. reported similar results for prostate cancer, investigating the frequency of needed edits to the AI‐assisted automatic segmentations and quality of the adaptive plans.^6^

In addition to these important studies to evaluate system performance on clinical data, there is a need for studies to evaluate robustness and reproducibility to develop quality assurance guidelines for online ART. End‐to‐end (E2E) tests which evaluate the full online adaptive planning process are useful to verify operation and stability of the clinical treatment process,^3^, ^7^, ^8^ and should include assessment of auto‐segmentation and plan adaptation—two critical steps of online ART. However, tools and processes for efficient and straightforward E2E of online ART are lacking. Deformable phantoms present a means for E2E tests, but are typically site‐specific,^8^ not widely available, and may not represent anatomically‐realistic deformations. Here, we present a comprehensive E2E process and results focusing on testing the reproducibility and robustness of the online adaptation process on the Ethos system. The goal of this work is to develop an E2E test strategy and determine uncertainty bounds for online segmentation and adaptive planning of the Ethos system to help establish action levels for the test. Our design goals for the E2E test strategy were to ensure it can be implemented at any clinic using only open source tools and is therefore easy and economical to realize. Rather than a deformable phantom, we developed a procedure to employ a deformed image of a rigid phantom which can be used with any number of anthropomorphic phantoms available in the market.

## 2 MATERIALS AND METHODS

This study involved an E2E procedure which utilized the following workflow: (1) a CT scan of an anthropomorphic pelvis phantom was taken; (2) synthetic deformations were applied to the CT image of the pelvis phantom near the area of the planning target volume (PTV); (3) the synthetically deformed datasets were used as the planning CT to generate reference plans; (4) a CBCT of the non‐deformed phantom was taken on the Ethos system, and the system adapted the deformed anatomy to the CBCT and automatically generated contours on the CBCT scan; (5) after the auto‐segmentation step, we either accepted the contours as generated or corrected them (explained further below); (6) scheduled (original reference plan re‐computed on the CBCT anatomy) and adapted (re‐optimized) plans were generated; and (7) adapted plan was evaluated relative to the reference plan. Each of these steps is explained in greater detail in the following sections.

### 2.1 Generating the synthetically deformed planning CT

A simulation CT scan of a CIRS Model 801‐P pelvis phantom (CIRS, Norfolk, VA) was acquired on a CT simulator, with baseline contouring of the following regions of interest: prostate, seminal vesicles, bladder, rectum, bowel, femoral heads, and clinical target volume (CTV). The pelvis phantom was chosen as it is available to us and has clearly defined and realistic organ boundaries.

The clinical online adaptive process of the Ethos system can only be used with a CBCT acquired by the system during a treatment session. In other words, an artificially‐deformed CBCT cannot be “injected” into the online adaptive process. To get around this limitation, we chose to deform the planning CT, and then image the undeformed phantom with CBCT. The planning CT used as the basis for creating the reference plan was generated by applying a synthetically generated deformation to this simulation CT in order to introduce deformed anatomy at the time of planning. By imaging the (undeformed) phantom with CBCT, the known, applied deformation could then be recovered by the Ethos registration and auto‐segmentation process.

To generate the synthetic deformation, a three‐dimensional Gaussian deformation was randomly located within an inscribed rectangular parallelepiped of size 22.3 mm left/right, 10.6 mm anterior/posterior, 62 mm superior/inferior within the PTV to ensure that deformations were centered near the target. A Gaussian deformation was selected as it has a known form which can be used as a ground truth and which can be easily implemented using a variety of off‐the‐shelf deformation and image analysis packages. While there are more realistic deformations that could be applied, such as implementing sliding organ boundaries or using more complex deformation basis functions, a Gaussian deformation ensures the developed E2E process can be implemented reproducibly at any clinic while still providing anatomically realistic deformations of the soft tissue within the pelvis for testing. The magnitude of deformation at the center of the Gaussian was randomly sampled between magnitudes of 10 and 20 mm. Standard deviations (falloff of the Gaussian deformation from the center) were randomly sampled between 15 and 25 mm. Deformations were generated and applied using the open source Plastimatch software (v 1.8.0, http://plastimatch.org/).

**FIGURE 1:**
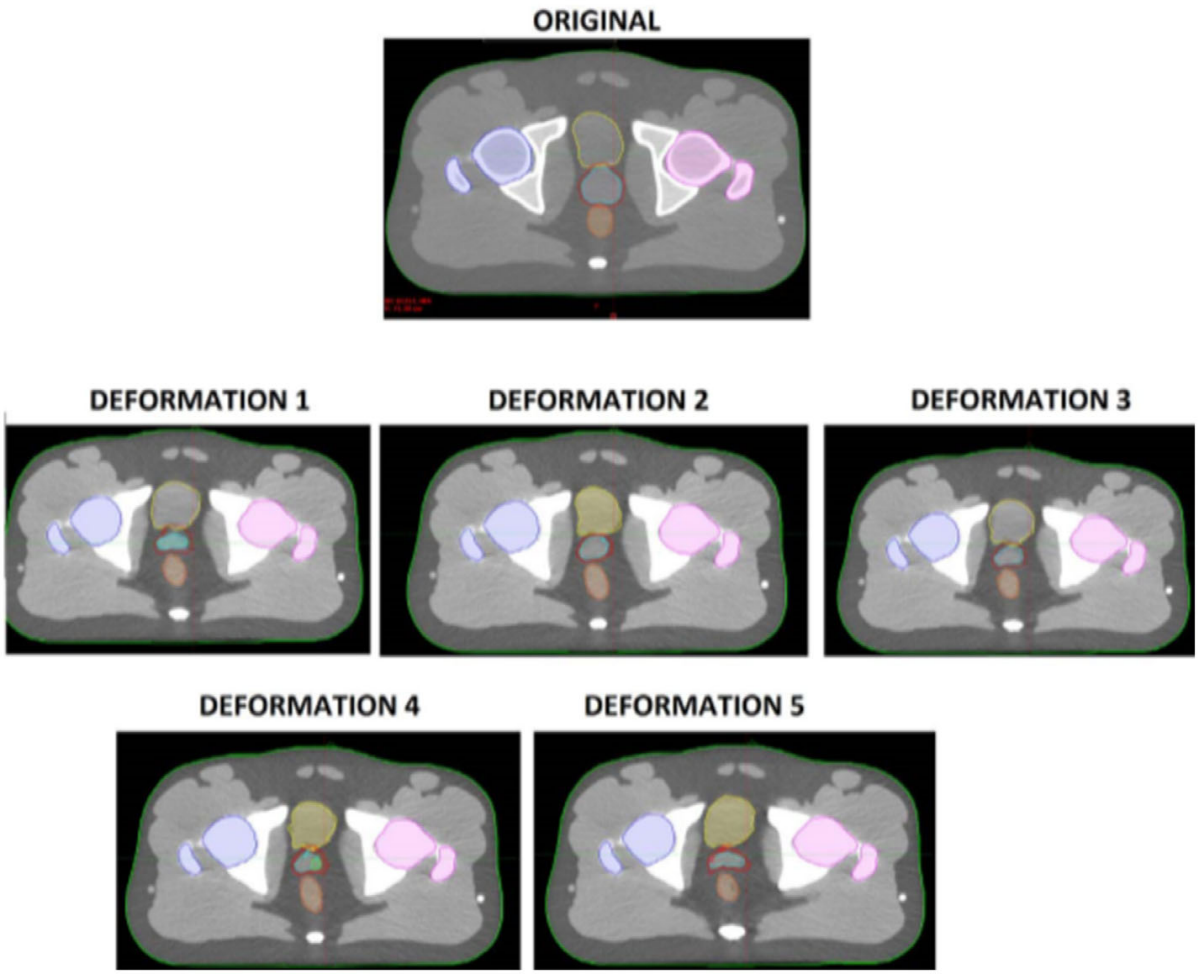
Original and synthetically deformed geometries used in this study. Contours shown: body (green), bladder (yellow), left femur (pink), right femur (blue), prostate (teal), seminal vesicles (light green), rectum (orange), PTV (red)

Using a single deformation repeatedly in an end‐to‐end test may bias the test results if the test performance depends heavily on the deformation applied. To study this effect and to trigger plan adaptation, five separate synthetic Gaussian deformations were generated and separately applied to the original CT image of the pelvis phantom, generating five separate deformed images. Table 1 gives the parameters for each of the five deformations applied, and each of the deformations is visually depicted in Figure 1.

**TABLE 1:** Gaussian parameters used for synthetic deformations applied to the initial planning computed tomography (CT) image

	Magnitude (mm)			Center (mm)			
**Patient**	***X***	***Y***	***Z***	***X***	***Y***	***Z***	Standard deviation
1	10.9	10.9	10.9	−13.4	119.6	707.7	18.7
2	−14.2	14.2	−14.2	−21.1	118.6	700.6	29.4
3	18.8	−18.8	−18.8	−6.2	119.8	708.1	15.6
4	−17.8	−17.8	17.8	−3.2	116.4	707.3	29.3
5	10.9	−10.9	10.9	−17.3	124.7	699.8	15.8

### 2.2 Treatment planning and delivery

For each synthetic deformation applied to the CT dataset, a corresponding patient (a total of five patients) was created in Ethos (v1.0, Varian Medical Systems, Palo Alto, CA.), and a plan was generated using the template shown in Table 2 to simulate a standard prostate treatment. For each patient, Ethos auto‐segments the structures listed in Table 2 using a proprietary algorithm, and then generates three intensity‐modulated radiation therapy (IMRT) and two volumetric modulated arc therapy (VMAT) plans. We began our study by selecting the IMRT plan and the VMAT plan that best met our planning goals.

**TABLE 2:** Planning constraints used in generating Ethos reference plans. Rx = prescription dose

**Total Rx**	**Total fractions (per patient)**
	**26**
**Region of interest**	**Constraint**
Planning target volume*	*D*98% > = 100% of Rx
	*D*_max_ < = 110% of Rx
Bladder	*V*35 Gy < 50%
	*V*57 Gy < 25%
Rectum	*V*35 Gy < 35%
	*V*57 Gy < 17%
Bowel	*V*45 Gy < 150cm^3^
Left femur	*V*45 Gy < 10%
Right femur	*V*45 Gy < 10%

* PTV = prostate + 5 mm.

^+^A prescription dose of 2.5 Gy/fraction to 70 Gy was used. However, only 26/28 fractions were used in the study.

For each patient, a 28 fraction plan was created but only 26 adapted and delivered. Three fractions each for the IMRT and VMAT plans were delivered without making any changes to the auto‐generated contours to assess the performance of the Ethos auto‐contouring algorithms. In the remaining 20 fractions, contours were corrected manually by a single observer, as would be done in clinical practice (10 fractions each for IMRT and VMAT plans). Because the E2E test is expected to be performed by a medical physicist, for this study a single medical physicist performed all the contour adjustments and approvals.

Each fraction was then delivered, and the scheduled and adapted plan parameters were recorded. In total, 130 fractions were delivered (26 fractions times 5 patients total).

### 2.3 Data collection and analysis

To evaluate variability of the AI‐based auto‐segmentation algorithm (AI contours) and manual correction process (manually corrected contours), the consistency of contours across multiple runs was measured. For each set of repetitions within each variable (plan type, deformation instance, and contour correction type), contour variability was measured in the following manner. First, a consensus contour was generated using STAPLE.^9^ Each organ contour for each repetition was then compared to the consensus contour for that set of repetitions. Contour comparisons included volume overlap as measured by Dice similarity coefficient and a distance metric (mean symmetric surface distance). The distribution of each metric across all repetitions was calculated to estimate variability.

A one‐way analysis of variance (ANOVA) was performed on the Dice similarity coefficient distributions to evaluate the impact of each synthetic deformation on contour variability. ANOVA was conducted separately for each region of interest (ROI). Based on the results of this ANOVA, 95% confidence intervals for the distribution of contour variability (separately, for Dice similarity coefficient and mean symmetric surface distance) were computed for each ROI.

## 3 RESULTS

### 3.1 Contour variability

**FIGURE 2:**
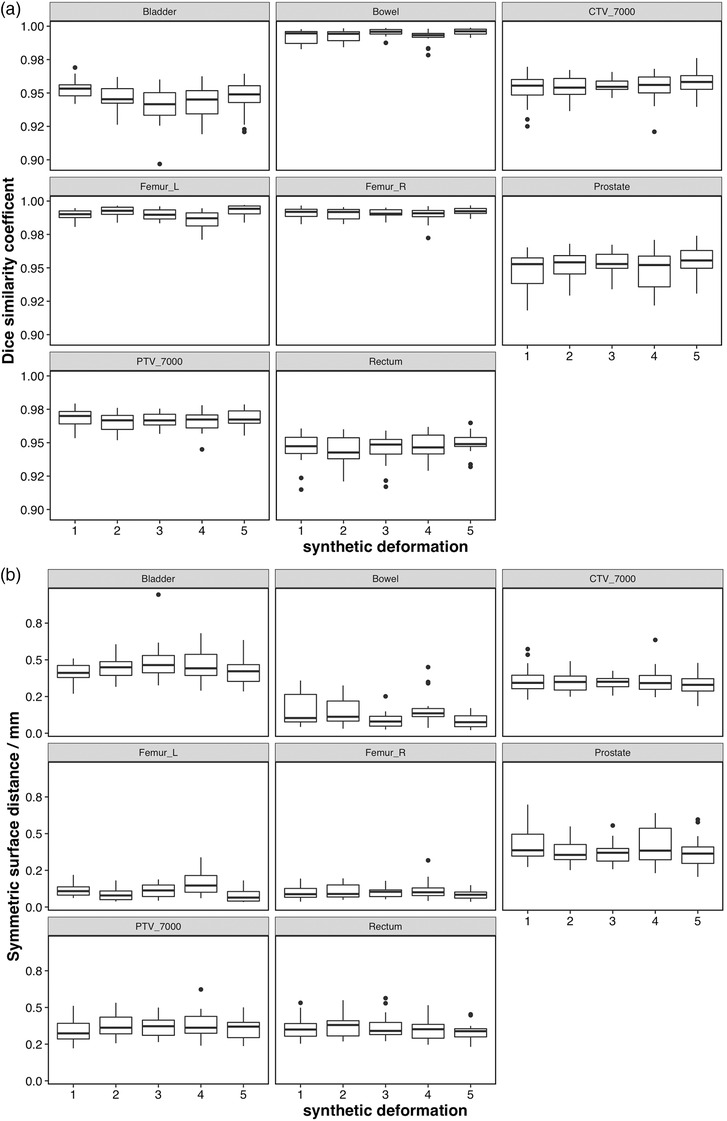
Intra‐observer repeatability of the manually‐corrected contouring process, all regions of interest, for all synthetic deformations. (a) Top: Dice similarity coefficient. (b) Bottom: mean symmetric surface distance

**FIGURE 3:**
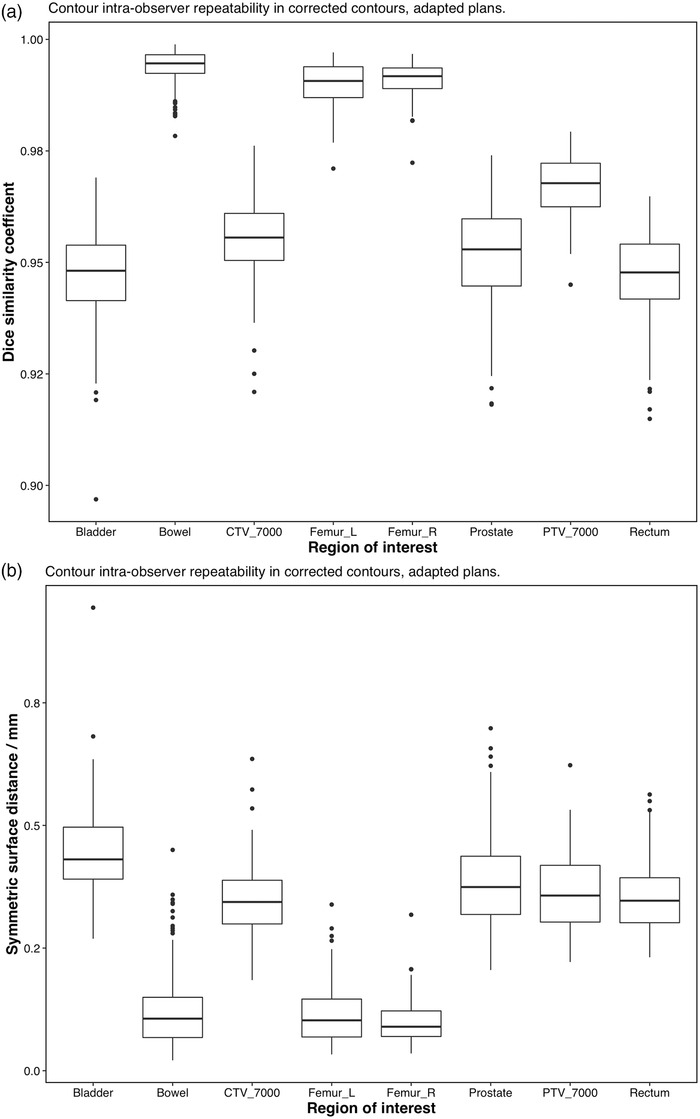
Intra‐observer repeatability of the manually‐corrected contouring process, all regions of interest, averaged over all deformations. (a) Top: Dice similarity coefficient. (b) Bottom: mean symmetric surface distance

Figure 2 shows the contour variability after manual correction for all five deformations applied for each ROI listed in Table 2. Figure 3 shows the same results, averaged over all five deformations. Dice similarity coefficient has a known dependence on volume and shape of the organ,^10^ but assessing both the Dice and distance measures, repeatability of the online contouring process varied with organ. Despite the known dependence of Dice similarity coefficient on shape and volume, Figure 3 shows that the organ‐specific performance as measured by Dice similarity and distance measures generally agree, with the repeatability and overall accuracy for bowel and femoral heads being the highest.

The results of one‐way ANOVA for Dice similarity coefficient are shown in Table 3. ANOVA for the mean symmetric surface distance gave similar results and are not shown. ANOVA showed that variations between different deformations were not significantly different than repeat measurements, except for the bowel contour (*p* = 0.026). This result can also be observed in Figure 2. Thus, all deformations for each ROI can reasonably be grouped together to generate an estimate of test uncertainty, as shown in Figure 3. Because the data shown in Figure 3 include all deformations, this figure can be used to estimate that uncertainty bounds that one might expect from a random deformation. In general, the AI‐assisted online contouring process showed a high degree of consistency among fractions (Dice > 93% for the vast majority of data, all mean surface distances < 1.0 mm).

**TABLE 3:** ANOVA evaluating the impact of synthetic deformation on contour variability and confidence intervals for Dice similarity coefficient, manually‐corrected contours

	*p* Value	Lower 95% CI, DSC	Upper 95% CI, DSC	Lower 95% CI, mSSD (mm)	Upper 95% CI, mSSD (mm)
Bladder	0.081	0.92	0.96	0.3	0.6
Bowel	0.026^*^	0.98	1.00	0.0	0.4
CTV_7000	0.095	0.93	0.97	0.2	0.5
Femur_L	0.71	0.98	1.00	0.0	0.3
Femur_R	0.51	0.98	1.00	0.0	0.2
Prostate	0.203	0.92	0.97	0.2	0.6
PTV_7000	0.87	0.95	0.98	0.2	0.5
Rectum	0.14	0.92	0.96	0.3	0.5

*Note*: Asterisk (*) denotes significance at the 0.05 level. DSC, Dice similarity coefficient; mSSD, mean symmetric surface distance.

Confidence intervals were also estimated directly from the distributions over the fractions and deformation plans, and these values are reported in Table 3.

**FIGURE 4:**
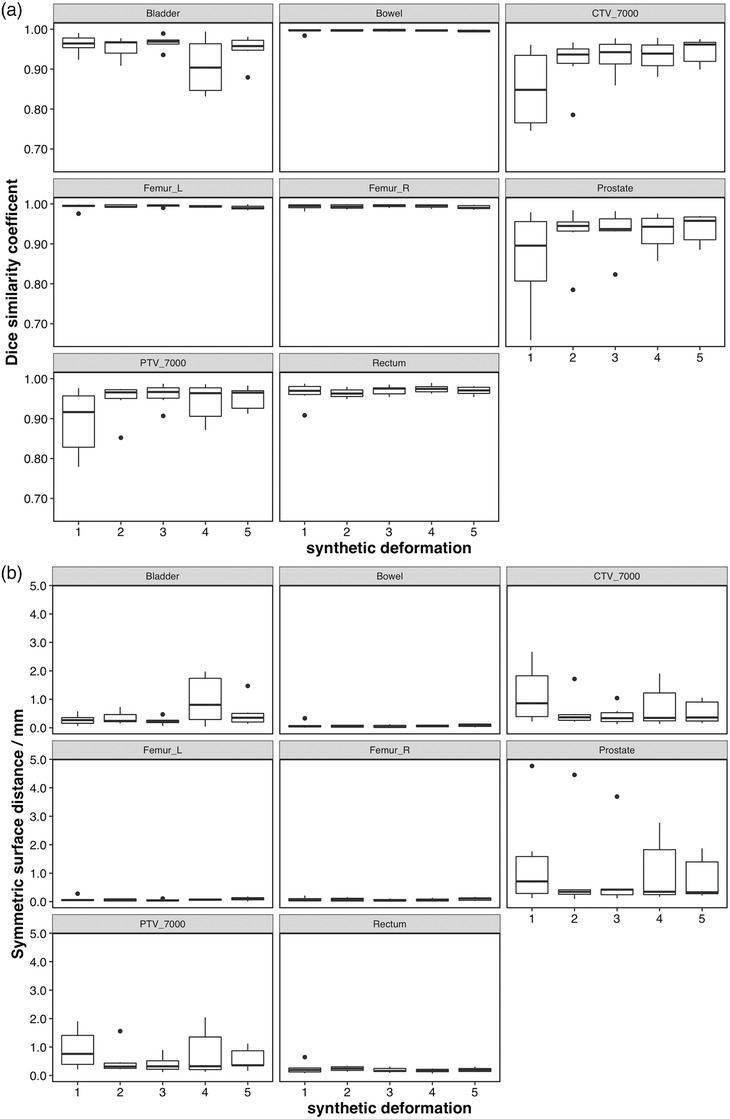
Intra‐observer repeatability of the artificial intelligence (AI) contouring (no manual correction) process, all regions of interest, for all synthetic deformations. (a) Top: Dice similarity coefficient. (b) Bottom: mean symmetric surface distance

**FIGURE 5:**
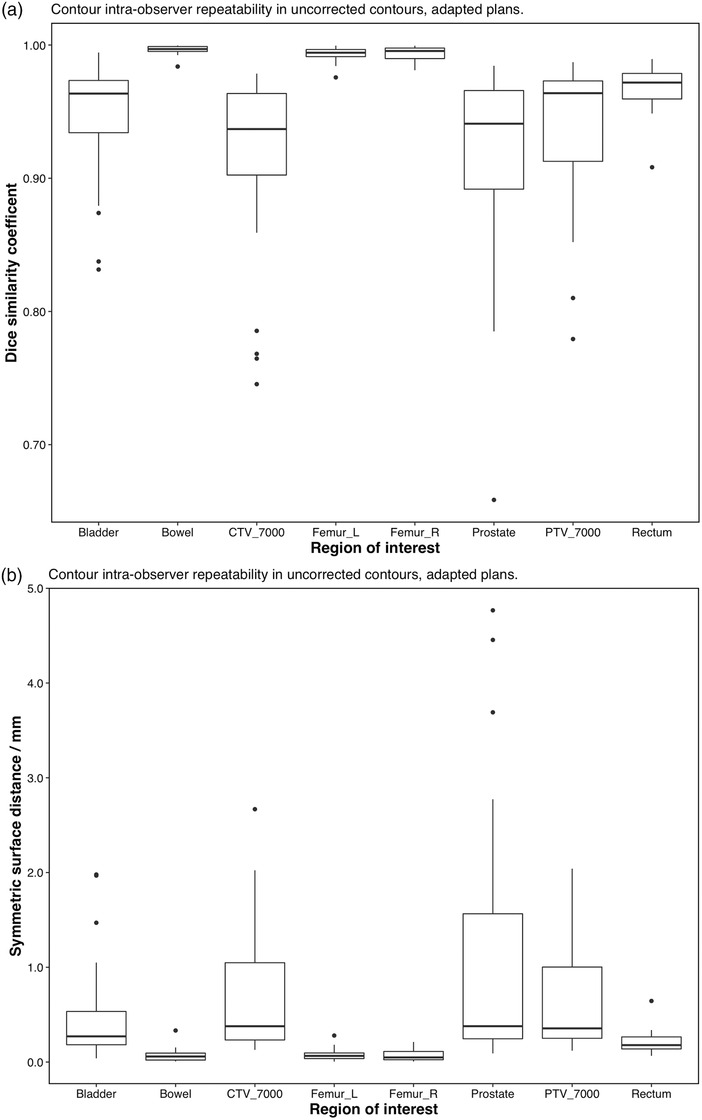
Intra‐observer repeatability of the artificial intelligence (AI) contouring (no manual correction) process, all regions of interest. (a) Top: Dice similarity coefficient. (b) Bottom: mean symmetric surface distance

Figures 4 and 5 show the contour variability results of the AI contours (without manual correction). Overall, there was more variability across the various synthetic deformations for bladder and target structures than with manual correction. However, the repeatability of bowel and femurs was higher for AI contouring alone than with manual correction. Table 4 shows the results of ANOVA for the AI contours, which showed one structure (CTV_7000) with statistically significant (*p* = 0.013) impact of synthetic deformation on the variability. Confidence intervals are also reported in Table 4.

**TABLE 4:** ANOVA evaluating the impact of synthetic deformation on contour variability and confidence intervals for Dice similarity coefficient, artificial intelligence (AI) contours

	***p* Value**	**Lower 95% CI, DSC**	**Upper 95% CI, DSC**	**Lower 95% CI, mSSD (mm)**	**Upper 95% CI, mSSD (mm)**
Bladder	0.195	0.84	0.99	0.1	2.0
Bowel	0.98	0.99	1.00	0.0	0.2
CTV_7000	0.013*	0.76	0.98	0.1	2.2
Femur_L	0.56	0.98	1.00	0.0	0.2
Femur_R	0.80	0.98	1.00	0.0	0.2
Prostate	0.10	0.75	0.98	0.1	4.5
PTV_7000	0.10	0.80	0.99	0.1	1.9
Rectum	0.23	0.94	0.99	0.1	0.4

*Note*: Asterisk (*) denotes significance at the 0.05 level. DSC, Dice similarity coefficient; mSSD, mean symmetric surface distance.

### 3.2 Adapted plans

**FIGURE 6:**
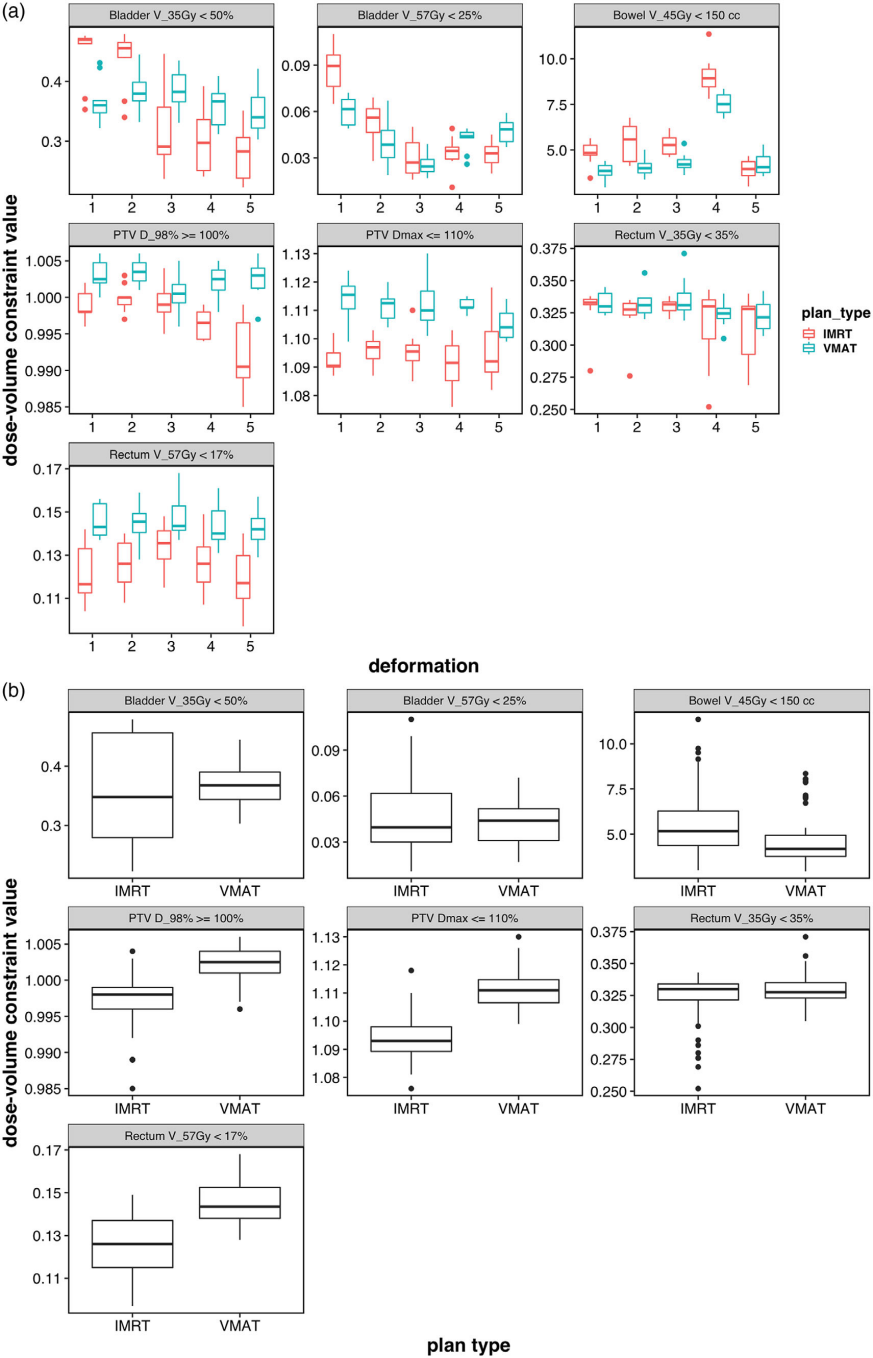
Repeatability of dose–volume values for adapted plans. (a) Top: for each synthetic deformation. (b) Bottom: grouped (all deformations together)

Action levels for adapted plans were quantified by plotting delivered adapted plan parameters for all five deformations applied for each organ listed in Table 2. These data are plotted in Figure 6a, and is broken down by plan type, with IMRT in red and VMAT in green. A one‐way ANOVA was performed on these data, which showed significant variation between plan types for several constraints. Thus, all deformations pertaining to each ROI can be analyzed together, as shown in Figure 6b, provided that the analysis is separated by plan type. As before, because the data shown in Figure 6b are across all deformations, this figure can be used to estimate that uncertainty bounds that one could expect from a random deformation applied in this phantom. Table 5 shows the 95% confidence intervals for these dose–volume values in Figure 6b.

**TABLE 5:** 95% confidence intervals for dose–volume constraint values, adaptive plans. Rx = prescription dose

**Region of interest**	**Constraint**	**Lower 95% CI, value**	**Upper 95% CI, value**
Planning target volume	*D*98% > = 100**%** of Rx	99**%**	100**%**
	*D*_max_ < = 110**%** of Rx	108**%**	111**%**
Bladder	*V*35 Gy < 50**%**	23**%**	48**%**
	*V*57 Gy < 25**%**	1.7**%**	9.9**%**
Rectum	*V*35 Gy < 35**%**	27**%**	34**%**
	*V*57 Gy < 17**%**	10**%**	15**%**
Bowel	*V*45 Gy < 150 cm^3^	3.39 cm^3^	8.05 cm^3^

In general, adapted plan dose–volume values were within a few percentage points of planning goals. VMAT plans achieved slightly improved target coverage while IMRT plans slightly improved normal tissue sparing for rectum and bladder, although all adaptive plans met the baseline planning constraints.

## 4 DISCUSSION

In general, the online contouring process (auto‐segmentation followed by manual correction) showed a high degree of consistency among fractions (Dice generally > 93%, all mean surface distances < 1.0 mm). Repeatability of this process varied with organ. Performance (both repeatability and accuracy) for bowel and femoral heads was highest. This is likely due to these structures being farthest from the region of largest deformation, which was near the prostate.

For AI‐based auto‐segmentation alone, repeatability and accuracy were high for femoral heads, bowel, and rectum. As the observer did not have access to the ground truth when correcting the AI‐based contours, in some cases (rectum, femoral heads), the AI‐based contours outperformed the manually‐corrected ones. Performance in the prostate (and related target volumes) and bladder demonstrated a need for manual correction. Since the conduct of this study, a maintenance update of the Ethos software (v1.1) has been released which allows for rigid transfer of the target volume. The results of this study suggest that this functionality may be useful in reducing the need for manual intervention to correct prostate target volumes, although this will need to be verified experimentally.

Compared to the results for auto‐segmentation, there was somewhat more variability between the five different deformations for the dose–volume values. One explanation is that the different deformations reconfigure the geometry between the targets and organs at risk, which introduces higher variability in the dose–volume value for each target and organ. Still, overall, the repeatability of adaptive planning was high as all constraints could be met despite the large deformation in the target and surrounding organs at risk. Similar results have been shown in clinical data in the pelvis and other body sites.^4^, ^5^, ^6^

The selected study design had some limitations. While a Gaussian deformation presents visually and anatomically plausible deformations, synthetic deformations do not completely represent ground truth, realistic deformation of anatomy. However, since the goal of this study is to assess repeatability and robustness, and to assist in setting baseline action levels for E2E testing of an online ART platform, our study design did not intend to perfectly match anatomically realistic deformation but instead prioritized an economical approach that can be completely implemented with open source tools and no coding. Additionally, repeating this study with anthropomorphic phantoms in a variety of body sites would allow for site‐agnostic action levels to be generated. This is reserved for future work.

## 5 CONCLUSION

This work assessed the repeatability of the AI‐assisted auto‐segmentation and adaptive planning process. A straightforward and efficient process was developed and used to quantify a set of organ‐specific contouring and dosimetric action levels to help establish uncertainty bounds for an end‐to‐end test on the Varian Ethos system. Our procedure can be deployed worldwide using a simple phantom.

## AUTHOR CONTRIBUTION

All authors were involved with the initiation, design, and conduct of the study. John W. Chapman, Bin Cai, and Geoffrey D. Hugo contributed to writing and editing the manuscript. All authors were involved in reviewing and approving the manuscript.

## CONFLICT OF INTEREST

The authors declare that there is no conflict of interest that could be perceived as prejudicing the impartiality of the research reported.
